# Reorganization of the Action Observation Network and Sensory-Motor System in Children with Unilateral Cerebral Palsy: An fMRI Study

**DOI:** 10.1155/2018/6950547

**Published:** 2018-07-25

**Authors:** Giuseppina Sgandurra, Laura Biagi, Leonardo Fogassi, Elisa Sicola, Adriano Ferrari, Andrea Guzzetta, Michela Tosetti, Giovanni Cioni

**Affiliations:** ^1^Department of Developmental Neuroscience, IRCCS Fondazione Stella Maris, Calambrone, Pisa, Italy; ^2^Department of Clinical and Experimental Medicine, University of Pisa, Pisa, Italy; ^3^Laboratory of Medical Physics and Biotechnologies for Magnetic Resonance, IRCCS Fondazione Stella Maris, Calambrone, Pisa, Italy; ^4^Department of Neuroscience, University of Parma and Istituto Italiano di Tecnologia (RTM), Parma, Italy; ^5^IRCCS S. Maria Nuova Hospital, Reggio Emilia, Italy; ^6^University of Modena and Reggio Emilia, Modena, Italy

## Abstract

Little is known about the action observation network (AON) in children with unilateral cerebral palsy (UCP). Using fMRI, we aimed to explore AON and sensory-motor network (SMN) in UCP children and compare them to typically developed (TD) children and analyse the relationship between AON (re-)organization and several neurophysiological and clinical measures. Twelve UCP children were assessed with clinical scales and transcranial magnetic stimulation (TMS). For the fMRI study, they underwent a paradigm based on observation of complex and simple object-manipulation tasks executed by dominant and nondominant hand. Moreover, UCP and TD children carried out a further fMRI session to explore SMN in both an active motor and passive sensory task. AON in the UCP group showed higher lateralization, negatively related to performances on clinical scales, and had greater activation of unaffected hemisphere as compared to the bilateral representation in the TD group. In addition, a good congruence was found between bilateral or contralateral activation of AON and activation of SMN and TMS data. These findings indicate that our paradigm might be useful in exploring AON and the response to therapy in UCP subjects.

## 1. Introduction

Functional representation of actions, either observed or performed or even imagined, relies on the human action observation network (AON), constituted by the premotor, inferior frontal, parietal, and temporal regions. Its functionality is crucial for action understanding and for subserving imitation by observation of new motor skills [1, 2].

Some neurophysiological studies exploring the presence and functionality of AON networks in children have suggested that maturation of AON has an age-related course from a more bilateral to a more lateralized representation, indicating physiological plasticity [3–5]. These properties are very meaningful, and it would be important to know if similar mechanisms could also be observed in pathological conditions such as unilateral or asymmetrical early brain injuries in children with unilateral cerebral palsy (UCP). It has been extensively demonstrated that the type of lesion and reorganization, studied through functional magnetic resonance imaging (fMRI) and transcranial magnetic stimulation (TMS), of the central nervous system have an impact on severity of upper limb deficits [6, 7]. Types of lesion, underlying UCP, are often categorized into three groups, according to location and timing of insult: type I (prenatal): malformations or 1st and 2nd trimester patterns, presumed to occur in utero such as lissencephaly, focal cortical dysplasia, unilateral schizencephaly; type II (perinatal): periventricular white matter lesions mainly occurring in the early 3rd trimester and often in preterm born infants such as periventricular leukomalacia (PVL); type III (connatal): cortical or deep grey matter lesions that occur towards the end of gestation, that is, around term age, such as infarcts in the territory of the middle cerebral artery (MCA) [7–9].

Previous studies have shown that children with type II lesions demonstrated better upper limb sensorimotor functioning, compared to children with type III lesions [10, 11]. Regarding type of motor reorganization, there are two main types of (re-)organization: ipsilesional and contralesional [12]. In adults with stroke and in some children, the main mechanism for reconnection of the motor cortex to spinal cord consists of (re-)organization within the ipsilesional cortex. This mechanism is based on partial sparing of the primary motor cortex or on the possibility that functions may be taken over by intact nonprimary motor areas within the damaged hemisphere (ipsilesional (re-)organization). However, when lesions occur at an early development stage, either during intrauterine life or soon after birth, a different mechanism can be observed. This is based on the persistence of a significant component of monosynaptic fast-conducting ipsilateral motor projections, normally withdrawing within the first months of life, that may be permanently maintained if brain damage occurs early in life [6, 13, 14]. In this case, the unaffected hemisphere directly controls both upper limbs, giving rise to a pattern of reorganization unknown in adult pathologies (contralesional (re-)organization). It has been extensively demonstrated that ipsilesional motor projection is definitely correlated to better motor outcomes, measured by functional scales (e.g., Melbourne Unilateral Upper Limb Measurement [15] and Assisting Hand Assessment [16]), than contralesional reorganization [11]. The sensory system generally follows an ipsilesional reorganization, but when a dissociation of sensorimotor representation occurs, that is, a contralesional reorganization for motor function and an ipsilesional reorganization for sensory one, quality of motor function is usually more affected [6].

Regarding AON in UCP children, Dinomais et al. [17] have shown, in eighteen UCP patients, aged 7–21 years, that observation at rest of a simple opening-closing hand movement performed by either the left or the right hand of an actor produces large bilateral activations in the occipito-temporo-parieto-frontal network, including most AON nodes. Moreover, a stronger ipsilesional activation of primary motor cortex (M1) was shown when they viewed movement of the hand corresponding to the affected one. Finally, observation of hand movement engaged motor execution networks regardless of degree of motor impairment.

The fMRI paradigm in this latter study was created around the observation of a simple movement without an object. We have developed an fMRI paradigm to explore AON based on the observation of simple and complex object-manipulation tasks executed by both dominant and nondominant hand. This fMRI paradigm has been already tested on healthy adults [18] and in a sample TD children [5].

The aim of this fMRI study was to explore AON and sensory-motor network (SMN) in UCP children in comparison to age-matched TD children and analyse the relationship between AON (re-)organization and several neurophysiological and clinical measures.

## 2. Methods

### 2.1. Subjects

Twelve UCP patients (6 with left UCP, age range = 6.2–16.3 y, mean age ± standard deviation (SD) = 10.3 ± 2.9 y) were enrolled in this study. The sample included seven males (age range = 7.5–14.5 y; mean age ± SD = 10.7 ± 2.6 y) and five females (age range = 6.2–16.3 y; mean age ± SD = 10.5 ± 4.0 y). All UCP children had IQ > 70.

Dataset from 12 healthy right-handed children and adolescents (6 M, 6 F; age range = 7.0–15.3 y, mean age ± SD(SD) = 10.6 ± 2.1 y) already described in a previous study [5] were used as age-matched controls (TD children).

All subjects and their parents gave written informed consent in accordance with protocol approved by the Ethics Committee of the IRCCS Fondazione Stella Maris.

### 2.2. Clinical Tests

All children were classified according to the House Functional Classification System (HFCS) for assessing upper limb function. HFCS consists of nine grades ranging from a completely excluded hand (grade 0) to a spontaneous and independent one (grade 8) [19, 20]. In addition, they were clinically assessed with two standardized function tests, Assisting Hand Assessment (AHA, Version 4.4) [16] and Melbourne Assessment of Unilateral Upper Limb Function (MUUL) [15], in order to evaluate assisting hand use during bimanual performance and upper limb movement capacity, respectively.

AHA is a standardized, criterion-referenced test based on observations of affected hand/arm used during a videotaped 15-minute play session with toys from the AHA test kit. Video scoring produces raw values ranging from 22 (low ability) to 88 (high ability) that are converted to scaled scores ranging from 0 to 100. AHA was administered and scored by a certified rater.

MUUL is a standardized tool for measuring quality of upper limb movement capacity during 16 criterion-referenced items representative of reach, grasp, release, and manipulation. Performance is videotaped and scored using criteria for rating qualities of movement range, fluency, and dexterity [15, 21]. Scores vary from 0 to 100%, the latter indicating best performance.

Presence or absence of mirror movements in the unaffected hand during voluntary unimanual movements of the affected hand was evaluated by consensus by two experienced child physical therapists (ES and EB, 30 and 8 years of experience in clinical evaluation of UCP), analyzing the videotapes of the standardized clinical tests.

### 2.3. Transcranial Magnetic Stimulation

TMS was performed using a mapping procedure as described in Borghetti et al. [22], by using a Magstim 200® device (Magstim Company Ltd., Whitland, Wales, UK) connected to a figure-eight coil (diameter: 11 cm). Both hemispheres were searched systematically for ipsilateral or contralateral motor-evoked potentials, during a monitored low-level contraction of abductor digiti minimi (ADM) muscles. TMS results were used globally to establish the type of reorganization of sensorimotor system. In particular, contralesional (CL) reorganization was used to indicate a reorganization involving the unaffected hemisphere, and ipsilesional (IL) for a reorganization involving the lesioned hemisphere. Data acquisition was conducted following the international guidelines for TMS in children for aspects of safety [23].

### 2.4. Experimental Design

In order to investigate the relation of AON with reorganization of SMN, two different fMRI paradigms were used.

**Figure 1:**
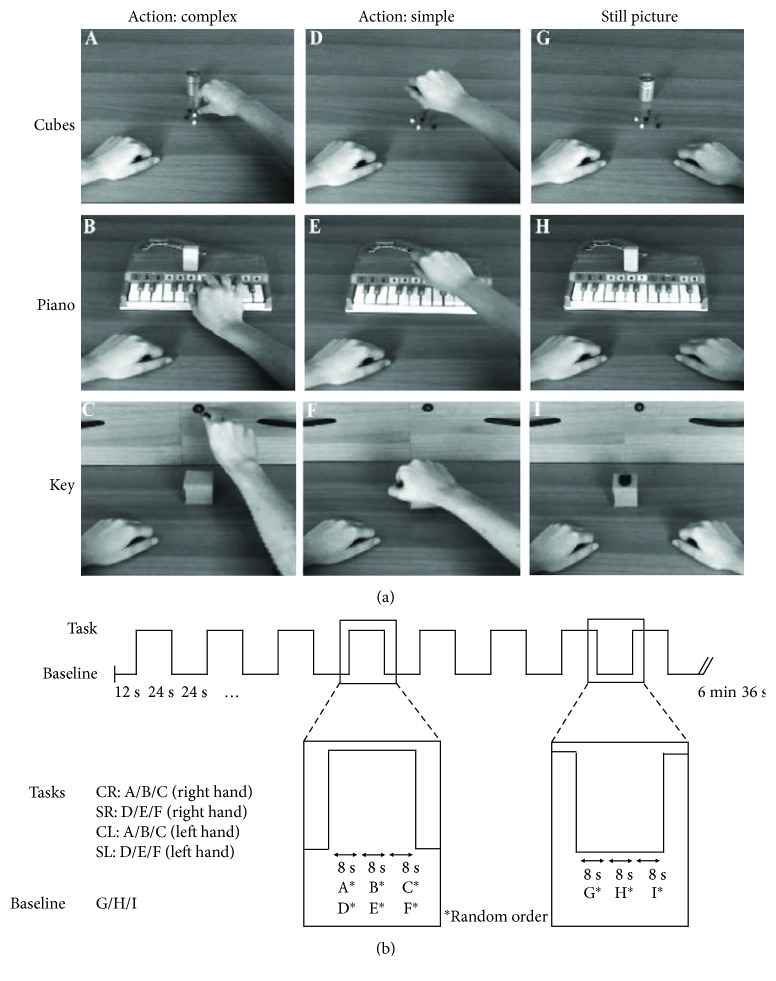
(a) Examples, taken from a single frame, of the six videoclips showing object manipulation performed by the right hand in three different contexts (“cubes,” “piano,” and “key”): three complex actions (A, B, C) and three simple actions with the same object (D, E, F). (G, H, I) Initial static frames of the corresponding action types, used as BASELINE conditions. (b) Diagram of the functional series presented to children: the block design comprises two TASK blocks for each of the four different conditions (CR, SR, CL, and SL) for complex (C) or simple (S) actions performed by the right (R) or left (L) hand, alternating with the same number of BASELINE blocks. Each block lasts 24 seconds and is composed of the random sequence of the 8-second videoclips of hand actions or still pictures of the resting hands. The presentation of the different conditions in the TASK blocks was completely randomized. Each functional series included four initial extra scans (12 s) to allow the stabilization of signal. Reproduced with permission (Copyright © 2015 John Wiley & Sons Ltd) from Biagi et al. [5].

AON was explored as described by Biagi et al. [5]. Eight-second videoclips in a first-person perspective of three simple and three complex hand actions, performed by dominant and nondominant hand, were presented (Figure 1(a)). The three complex actions were grasping little cubes and putting them into a box (cubes), performing a simple scale on a piano keyboard (piano), and grasping a key, putting it into a lock, and turning it (key). The three simple actions consisted of a whole hand grasping a small box performed in the same visual contexts of the complex actions, in order to match luminance, colour, and visual information. Videoclips of the same type (hand, complexity) in the three contexts (cubes, piano, and key) were randomly combined to create four distinct conditions (TASKS), each lasting 24 seconds, corresponding to the presentation of a simple or a complex action performed by dominant or nondominant hand (simple-dominant (S-D), complex-dominant (C-D), simple-nondominant (S-ND), and complex-nondominant (C-ND)). The 24-second corresponding control condition (BASELINE) was created by combining a sequence of three still pictures of resting hands in the respective contexts. The paradigm of stimulus presentation was built on a block design scheme, with two blocks for each of the previous four TASKS intertwined with the same number of BASELINE blocks (16 blocks total). In each functional series, the order of TASKS blocks was randomly generated and four initial extra scans (dummies, 12 seconds) were added to allow for stabilization of signal, giving a total acquisition time of 6′36″ (Figure 1(b)). The AON experiment consisted of the acquisition of two functional series. Visual stimuli were presented through LCD goggles (Resonance Technology, USA). Subjects were asked to observe videos, staring at the middle of a screen. Gaze and attention to stimuli were continuously monitored using an eye tracker infrared camera mounted on goggles. Recorded eye movements were analysed to verify gaze and attention during AON stimuli.

For SMN localization, a block design paradigm was designed with both an active movement task (alternated hand opening and closing, MOTOR TASK) and a passive sensory task (palm and fingers passively brushed by an external operator by means of a wooden spatula, at a frequency of about 1 Hz, SENSORY TASK), in the same functional series. The series included eight blocks of 18 seconds each, alternating between motor and sensory tasks, each intertwined by an equivalent number of REST periods (REST-MOTOR TASK-REST-SENSORY TASK…). All subjects received detailed instructions before acquisition. They were asked to keep their eyes closed. During motor task, they were asked to repetitively open and close their hand at a frequency of 1 Hz; commands “move” and “stop” were given at the beginning and end of each block. During the sensory task and rest periods, they were asked simply to stay still. The examiner visually controlled the task performance. Each series included four initial extra scans (dummies, 12 seconds) for a total acquisition time of 5 minutes. Two sessions were performed, the first for the dominant hand and the second for the nondominant one, obtaining four conditions of interest (sensory-dominant (Sens-D), motor-dominant (Mot-D), sensory-nondominant (Sens-ND), and motor-nondominant (Mot-ND)).

During fMRI acquisitions, ambient scanner noise was constant and attenuated by ear plugs.

### 2.5. Imaging Acquisition and Processing

MR exams were performed on a 1.5 T MR scanner (HDx, GE Healthcare, Milwaukee, WI, USA). Standard MR protocol included FSE T2-weighted, SE T1-weighted, FLAIR, and DWI sequences. A whole brain, 3D high-resolution, T1-weighted series (FSPGR) was collected in an axial plane (TR/TE = 12.3 ms/2.4 ms; TI = 700 ms; voxel size = 1 mm^3^ isotropic) for anatomic localization of activated regions and delineation and description of lesions.

MRI anatomical findings were classified retrospectively according to literature [7, 9] into three main forms related to timing of lesion: type I, brain malformations (early malformative); type II, abnormalities of periventricular white matter (prenatal); and type III, cortical-subcortical lesions, mainly due to middle cerebral artery infarction (connatal).

The fMRI session included four series, two functional series for AON task (each lasting 6′36^″^) and two series for sensory-motor task (5′00″), one for each hand. Blood oxygenation level-dependent (BOLD) responses were registered by using an echo planar imaging gradient-echo sequence (GRE-EPI) with the following parameters: TE/TE = 3000/50 ms, FA = 90°, field of view (FOV) = 240 × 240 mm, matrix = 64 × 64, slice thickness = 5 mm.

Data preprocessing, performed using BrainVoyager QX Software Package (BV, Brain Innovation, Maastricht, the Netherlands), included mean intensity adjustment to compensate for interscan intensity differences, temporal interpolation and resample to compensate for slice-dependent time differences (sinc function), 3D motion correction (sinc interpolation), and high-pass temporal filtering (GLM-Fourier approach, two cycles/time course).

Functional data were coregistered on the three-dimensional anatomical T1-weighted images by using an affine alignment with the standard BV nine parameters (three for translation, three for rotation, and three for FOV scale). Anatomical datasets were in turn transformed into standard Talairach's Space [24].

In order to combine data from UCP children in a group analysis, and considering that TD children were all right-handed, we designated the left hemisphere as the hemisphere contralateral to the dominant hand (unaffected hand in UCP, right hand in TD) and the right hemisphere as the hemisphere contralateral to the nondominant hand (affected hand in UCP, left hand in TD). To do this, we flipped the right-left (x) direction [17] of both functional and structural T1-weighted images of children with left hemisphere lesions (right hemiplegia), in order to have all lesioned hemispheres on the right side of the brain and all unaffected hemispheres on the left side.

### 2.6. fMRI and Statistical Analysis

BOLD responses were analysed using the general linear model (GLM) approach, using the same number of regressors as conditions of interest. Each regressor was obtained by convolving a box-car function for each stimulation block with the standard Boynton hemodynamic response function [25]. Four regressors were selected both for the AON stimulus (S-D, C-D, S-ND, and C-ND) and for the SMN task (Sens-D, Mot-D, Sens-ND, and Mot-ND). In all analyses, six spurious movement regressors (outputs of the 3D motion correction procedure) were included in GLM.

Multisubject analyses were conducted using random-effects (RFX) GLM-based analysis, for both groups (UCP and TD) in order to identify a group representation of cortical activations and to detect possible differences between groups. Threshold of statistical maps was *p* < 0.05 Bonferroni-corrected, and a minimum cluster size of 150 mm^3^ was also applied. Group analyses were also used to reveal and define regions of interest (ROIs), to be selected subsequently in single-subject analysis.

For the AON stimulus, contrasted activity for all observed actions versus control condition was used (all TASKS > BASELINE) in order to identify possible differences in the representation of AON in UCP children with respect to their age-matched controls. Moreover, contrasted activities for observation of complex and simple actions performed by the dominant or nondominant hand versus control condition ((C-D + S-D) > BASELINE, (C-ND + S-ND) > BASELINE)) were also performed, in order to investigate hand identity properties in UCP children.

As in Biagi et al. [5, 18], a ROI analysis was conducted on activated areas in order to investigate possible differential responses to observation of both hands (laterality) and to observation of complex and simple actions (complexity). In particular, a 2 × 2 factorial design ANOVA was performed in specific areas (anterior intraparietal cortex (AIP), inferior temporal gyrus, MT, as control), considering as factors “hand” laterality (two levels: dominant and nondominant) and the “complexity” of action (two levels: simple and complex).

Finally, probabilistic functional maps were calculated to evaluate spatial consistency of activity patterns across subjects.

For the SMN task, two contrasts were employed, considering together sensory and motor stimuli of each hand, dominant or nondominant ((Sens-D + Mot-D) > REST and (Sens-ND + Mot-ND) > REST).

For the same contrasts, single-subject analyses were performed by using a fixed-effects (FFX) approach, with a lower, uncorrected statistical threshold (*p* < 0.001, minimum cluster size ≥ 150 mm^3^) in order to extract, from each participant, coordinates of foci and number of activated voxels of areas identified by multisubject group analysis. Average and variability (defined as standard deviation divided by mean: SD/mean × 100) across subjects were also calculated. Moreover, a laterality index (LI) was calculated by comparing the size of homologous areas in both hemispheres. In particular, for the AON task, LI was obtained by computing the ratio (*N*_DS_–*N*_nonDS_)/(*N*_DS_ + *N*_nonDS_), where *N*_DS_ and *N*_nonDS_ are the number of activated voxels in the dominant side of the brain (DS, hemisphere contralateral to dominant hand) and in the nondominant side (non-DS, hemisphere contralateral to nondominant hand), respectively. For the SMN task, a laterality index was also calculated specifically for the primary sensorimotor cortex (pSMC), using the same formula, but considering only the number of activated voxels in the two pSMCs.

For hemispheric dominance [26], we assumed a standard threshold of 0.20 in absolute value: LI > 0.20 dominance in the hemisphere contralateral to the dominant hand, LI < −0.20 dominance in the hemisphere contralateral to the nondominant hand, and −0.20 ≤ LI ≤ 0.20 bilaterality.

Statistical analysis and data fitting were performed via the software package OriginPro 9.0 (OriginLab Corporation). For linear data fit, Pearson correlation coefficient and equivalent *p* value were calculated. For data comparison of both groups (UCP and TD) and for data regarding the two hands and respective hemispheres, a nonparametric Mann–Whitney *U* test was used.

## 3. Results

**Figure 2:**
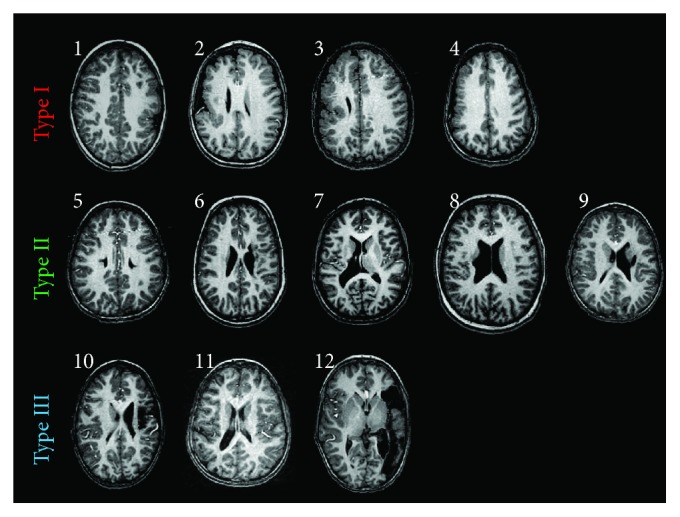
Representative slices of 3D T1-weighted images depicting the brain lesion of each UCP child. Numbers correspond to the ID of UCP children as reported in Table 1.

Table 1 shows demographic and clinical data of enrolled UCP children. According to type and timing of lesions, the sample was classified into three groups: the first one was composed of four children with early malformative lesions (type I), the second one of five children with white matter damage (type II) and the third group by three children with connatal stroke (type III). In all patients, except one, the lesion was strictly unilateral (see Figure 2). In patient 5, who had bilateral alterations on imaging, the more affected hemisphere was contralateral to the side of motor impairment.

Based on TMS results, all children with type I lesions showed a CL reorganization, while children with the other two types of lesion showed either CL or IL reorganization.

Mirror movements (MM) were present in all children with type I lesion, while they were variably present or not in children with the other two types of lesion.

In the fMRI experiment, all children were able to understand tasks and succeeded to collaborate and to maintain gaze in the middle of the screen for the AON task. For the SMN task, one child (number 4) in the UCP group was discarded from the following analysis because of the presence of excessive movement artefacts during functional acquisition, which were difficult to correct or compensate for.

### 3.1. AON in UCP and TD Children

As previously observed in TD children [5], also UCP children showed activation of areas belonging to the action observation network such as the inferior temporal gyrus (BA37), superior temporal sulcus (BA 22), anterior intraparietal sulcus (BA40-7), inferior parietal lobule (BA40), superior parietal lobule (BA7), precentral gyrus (dorsolateral, BA6-9 and BA6-4), and inferior frontal gyrus (BA45-47). As in TD children, further activations were found in visual and somatosensory areas and in the middle frontal gyrus.  Table 2 reports averages of Talairach's coordinates and of cluster sizes of activated areas, calculated across subjects of both groups, for the AON task.

**Figure 3:**
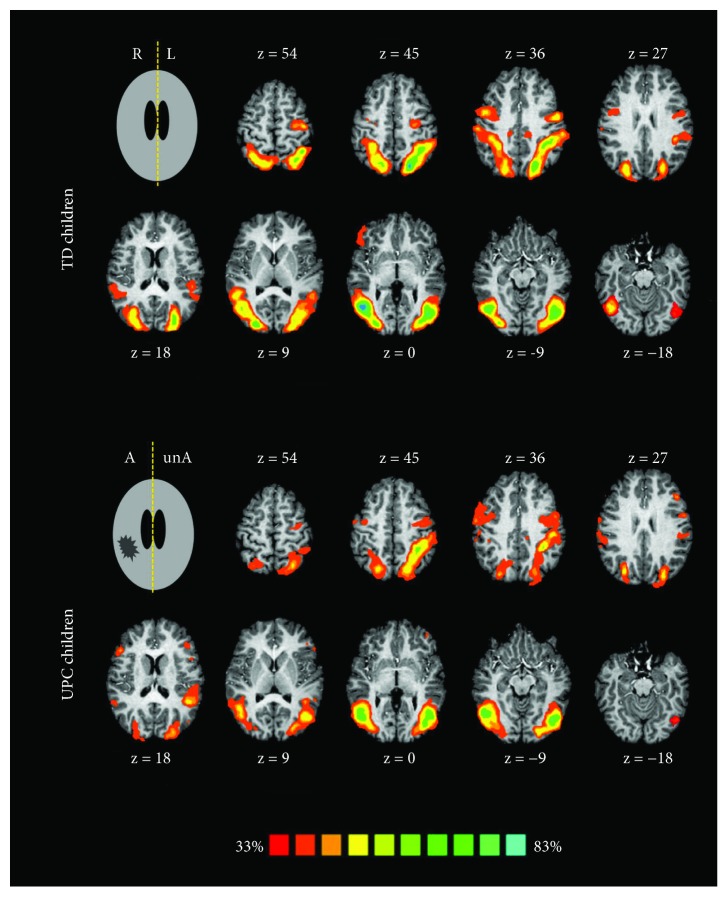
Probabilistic functional maps of the action observation circuit for the ALL TASKS > BASELINE contrast in TD children (as previously published in Biagi et al. 2015, top [5]) and in UCP children (bottom). Colour bar represents different levels of probability of activation for the action observation task from 33% (red, meaning that a brain region appeared in the map only if it was activated in at least 4 subjects) to 83% (cyan, equivalent to areas activated by more than 10 subjects). For each transversal slice, the *z* Tailarach's coordinate is indicated. On the right, we represent the left hemisphere for TD children (radiological convention; R = right, L = left) and the unaffected hemisphere, that is, the hemisphere contralateral to the unaffected hand, in UCP children (unA = unaffected, A = affected); on the left, the right hemisphere for TD and the hemisphere contralateral to the affected hand in UCP.

Similarly, Figure 3 shows probabilistic maps about the contrast of all TASKS > BASELINE for UCP and TD children, allowing for a comparison of results between both groups.

Pattern of activations of AON in TD children presented higher levels of probability with respect to the UCP group, suggesting a more reproducible network. This was also confirmed by variability analysis conducted at the level of single-subject data, where the coefficient of variation of number of activated voxels across subjects was overall significantly different between TD children (67%) and UCP children (89%) (Mann Whitney *U* test, *p* = 0.003).

Regarding features of stimuli (“hand” laterality and “complexity” of action), a 2 × 2 factorial design ANOVA revealed a significant effect only for hand identity property (“hand,” *p* < .001) in AIP of the hemisphere contralateral to the nondominant hand. However, no significant effects were found for the “complexity” factor (*p* = 0.23) and the interaction effect (“hand” × “complexity,” *p* = 0.28). No significant effect was found in either right or left MT, used as a control area.

### 3.2. Sensory-Motor Task in UCP and TD Children

BOLD responses to both sensory and motor tasks of both hands (contrasts: (Sens-D + Mot-D) > REST and (Sens-ND + Mot-ND) > REST) allowed for the identification of similar activity patterns in a number of cortical areas belonging to SMN in both groups.

In particular, the majority of subjects showed bilateral activations in the primary sensory motor cortex (pSMC, BA 1-2-3-4) and in inferior parietal lobule (BA 40). Other activations were found medially in the supplementary motor area (SMA), in a sector comprised between medial frontal gyrus and cingulate gyrus (BA 6-31), in the precentral gyrus of the hemisphere contralateral to the stimulated hand (BA 6), and in the cerebellar hemisphere ipsilateral to the stimulated hand. Differently from TD children, UCPs also showed activity in the insula (BA 13), contralaterally in the case of stimulation of the dominant hand, and bilaterally for the nondominant one.  Table 3 reports averages of coordinates and number of activated voxels, calculated across subjects for both groups.

### 3.3. Lateralization of AON and of pSMC in UCP and TD Children

Concerning AON, Figure 3 shows bilateral brain activation in TD children, as previously reported in Biagi et al. [5]. On the contrary, UCP children presented a mildly lateralized circuit in the hemisphere contralateral to the dominant hand. This finding was obtained by computing global lateralization indices, using number of voxels in the two hemispheres obtained by multisubject analysis (LI_TD_ = 0.01, LI_UCP_ = 0.23).

**Figure 4:**
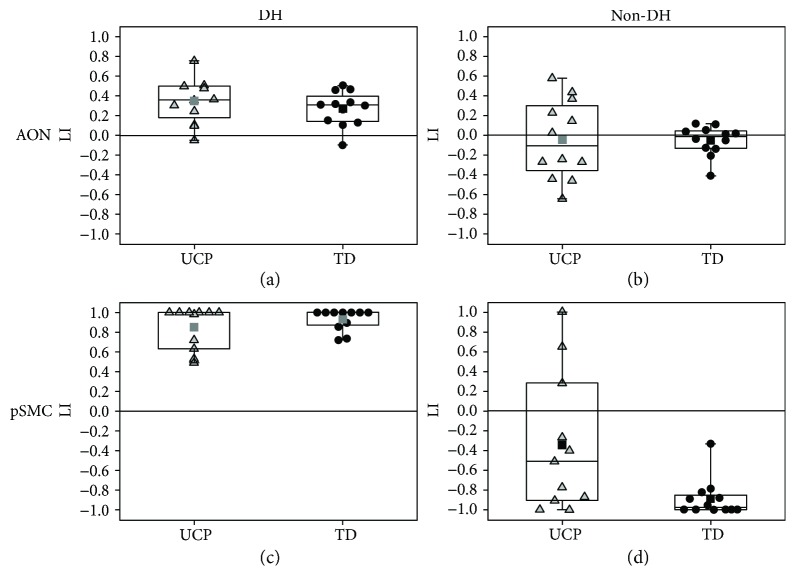
Box plots of the laterality indices of each single subject for AON (top row) of the dominant hand (DH) (a) and of the nondominant hand (non-DH) (b), as well as for the primary sensory-motor cortex, pSMC (bottom row), for the stimulation of the dominant hand (c) and of the nondominant hand (d). UCP children are indicated by light grey triangles, TD children by black circles. Each box is defined by the 25th and 75th percentiles. The whiskers are determined by the minimum and maximum values; the square indicates the mean value, while the line corresponds to the median value.

Regarding the two hands separately, Figure 4 reports data on lateralization indices obtained in each subject for both stimuli. For AON, LIs were calculated considering the whole neuronal circuit identified by multisubject analysis, while for SMN, LIs referred more specifically to lateralization of the primary sensorimotor cortex (pSMC).

Observation of simple and complex actions performed by the dominant hand ((C-D + S-D) > BASELINE) induced a slightly higher, but not significant, activation of contralateral hemisphere in UCP children (LI_UCP_ = 0.35 ± 0.22) with respect to the TD group (LI_TD_ = 0.27 ± 0.17) (*p* = 0.35, Figure 4(a)). LI mean values obtained from observation of actions performed by the nondominant hand ((C-ND + S-ND) > BASELINE) were very similar between the two groups (LI_UCP_ = −0.04 ± 0.40; LI_TD_ = −0.05 ± 0.15, *p* = 0.98) suggesting bilateral networks. However, in the UCP group there was a higher variability among children (LI_UCP_range = −0.64 ÷ 0.58; LI_TD_range = −0.40 ÷ 0.12), because single subjects presented very different lateralization indices. In particular, 4 subjects were lateralized to the hemisphere contralateral to the dominant hand, 6 subjects to the hemisphere contralateral to the nondominant hand, and only 2 subjects showed bilateral representation (Figure 4(b)). By comparing the two hands with an intragroup analysis, both groups presented significant differences in LI values between observation of the dominant and nondominant hand (*p* = 0.02 in the UCP group, *p* = 4 · 10^−4^ in TD).

For SMN, laterality indices of pSMC for stimulation of the dominant hand ((Sens-D + Mot-D) > REST) were similar between the two groups (LI_UCP_ = 0.85 ± 0.21, LI_TD_ = 0.93 ± 0.11, Figure 4(c)). On the contrary, there was a significant difference for values obtained with stimulation of the nondominant hand ((Sens-ND + Mot-ND) > REST; LI_UCP_ = −0.35 ± 0.7; LI_TD_ = −0.89 ± 0.19; *p* = 0.02; Figure 4(d)). Also for SMN, both groups presented significant differences in an intragroup analysis, when comparing LI values of both hands (*p* = 0.001 in UCP, *p* < 1 · 10^−4^ in TD).

### 3.4. Correlations between LI and Clinical Scores, Type of Lesion, and Reorganization in UCP Children

Due to great variability among subjects in the UCP group, a correlation analysis was performed between laterality indices and clinical scores, types of lesion, and type of reorganization.

**Figure 5:**
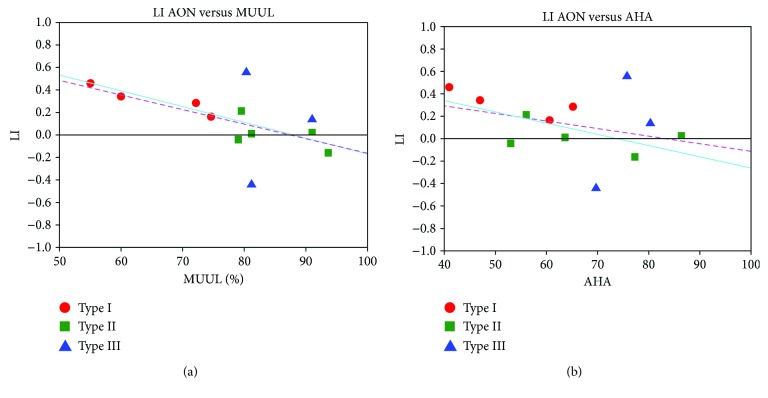
Lateralization index (LI) for the observation of all object-related actions versus BASELINE for each UCP child, plotted against his/her clinical scores (MUUL scale on panel a; AHA scale on b). Children were represented with different colours and symbols according to the classification of their lesions (type I = red circles, type II = green squares, type III = blue triangles). Data were fitted with a standard linear function. Considering all the subjects, the correlations are not significant (Pearson's value *R* = −0.55, *p* = 0.06 for MUUL; *R* = −0.34, *p* = 0.28 for AHA; pink dotted line). They become significant when only children with lesions of type I and type II are used in the fit (*R* = −0.90, *p* = 0.0007 for MUUL; *R* = −0.68, *p* = 0.04, for AHA; cyan solid line), due to big variability of data from children with type III lesion.

For the AON task and all TASKS > BASELINE contrast, LIs of single UCP subjects were plotted against their respective values of clinical scales (Figure 5).

A negative correlation was found between LI and percentage MUUL and AHA scores. This finding is not significant, considering all subjects with three types of lesions in linear fit (*p* = 0.06 for MUUL; *p* = 0.28 for AHA). On the other hand, it is significant if only children with type I and type II lesions are included in the analysis (*p* = 0.0007 for MUUL; *p* = 0.04 for AHA). The same type of analysis was repeated considering only LI values obtained from observation of actions performed by the nondominant hand ((C-ND + S-ND) > BASELINE). Data showed similar trends, but statistical analysis was not significant (*p* = 0.07 for MUUL, *p* = 0.08 for AHA). No significant correlations were found in the same analysis using LI values of pSMC for sensory-motor stimulation of nondominant hand and clinical scales.

**Figure 6:**
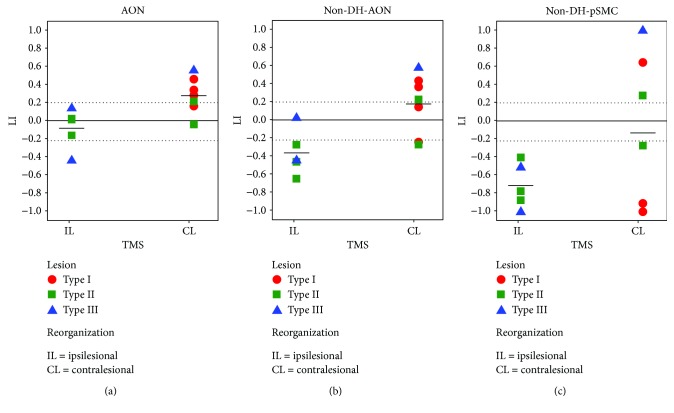
Box plots of LI values obtained by different contrasts (AON task: all TASK>BASELINE, panel a; AON task: (C-ND + S-ND) > BASELINE, panel b; and sensory-motor task: (Sens-ND + Mot-ND) > REST, panel c) in UCP children, grouped according to TMS data (CL = contralesional reorganization, IL = ipsilesional). As in Figure 5, children were represented with different colours and symbols with respect to the classification of their lesions (type I = red circles, type II = green squares, type III = blue triangles). Grey dotted lines represent the threshold value of |0.20| for hemispheric lateralization.

Figure 6 shows box plots of LI values of UCP children, grouped according to TMS results for different contrasts and different stimuli.

For all TASKS>BASELINE contrast of AON (Figure 6(a)), LI values of UCP children were significantly different when matched with the two types of reorganization revealed by TMS (*p* = 0.023, Mann–Whitney *U* test). In particular, children with IL reorganization at TMS (i.e., in the affected hemisphere) had bilateral AON activation (LI_IL_ = −0.08 ± 0.22), except for one case (number 10), who presented a marked lateralization to the affected hemisphere. On the contrary, children with CL reorganization at TMS (i.e., in the unaffected hemisphere) presented either bilateral activation or higher activation in the hemisphere contralateral to the dominant hand (LI_CL_ = 0.28 ± 0.20).

If the previous analysis is performed considering only observation of the nondominant hand (contrast: (C-ND + S-ND) > BASELINE, Figure 6(b)), both lateralization indices decrease accordingly ((LI_IL_ = −0.36 ± 0.25; LI_CL_ = 0.18 ± 0.33) and their differences continue to be statistically significant (*p* = 0.023, Mann–Whitney *U*-test).

Regarding SMN of the nondominant hand (Figure 6(c)), UCP children with a different reorganization at TMS presented different trends of LI values of pSMC, even if globally their differences were not statistically significant (*p* = 0.58). Children with IL reorganization at TMS showed a greater activation of pSMC in the affected hemisphere (LI_IL_ = −0.71 ± 0.25), in accordance to TMS. On the contrary, children with CL reorganization did not present a common behaviour, but rather a certain degree of variability (LI_CL_ = −0.13 ± 0.88). Three cases (numbers 12, 2, and 8) showed lateralization of pSMC in the unaffected hemisphere (LI > 0), while the other three have it in the affected hemisphere (LI < 0): one (number 7) presented bilateral representation of pSMC, and two subjects (numbers 1 and 3) showed discordant reorganization with respect to TMS, showing an evident lateralization of pSMC to the affected hemisphere.

Similar results were found when this analysis was performed using the mirror movements data as discriminating factor (Figure in Supplementary Materials (available here)) .

### 3.5. Comparisons between AON and SMC in UCP and TD Children

**Figure 7:**
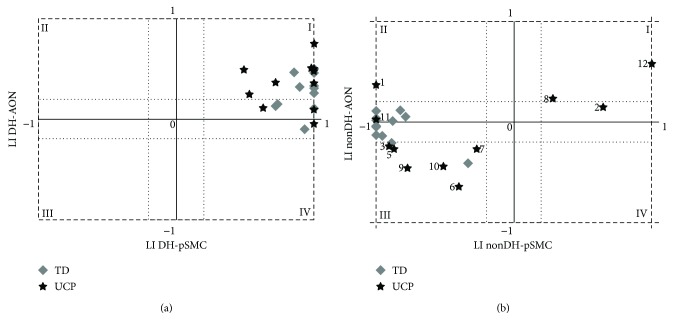
Four-quadrants charts of LI values of pSMC in the sensory motor task (abscissae) and of the AON (ordinates) of the two hands: dominant hand (DH) on panel a, nondominant hand (non-DH) on b. The first and third quadrants represent the congruence of the sign (both positive in the first, I; both negative in the third, III), while the second and the fourth represent the discordance of the sign (pSMC-negative and AON-positive in the second, II; pSMC-positive and AON-negative in the fourth, IV). TD children were represented by grey diamonds, UCP children by black stars. In the chart for the nondominant hand, labels on data of UCP children are used to identify subjects, according to Table 1.

In order to directly compare SMN and AON tasks, LI values were reported in a four-quadrants chart (SMN in *abscissae* and AON in *ordinates*), in which the first and third quadrants represent concordance of sign (both positive in the first, I, and both negative in the third, III), while the second and the fourth represent discordance of sign (one positive and one negative) (Figure 7).

In the case of stimuli performed by the dominant hand (Figure 7(a)), all subjects of both groups lie within the first or fourth quadrant, but very close to the zero axis, corresponding to a concordance between contralateral representation for pSMC and contralateral or bilateral representation for AON. Instead, scattered data were found in the case of stimuli performed by the nondominant hand (Figure 7(b)). All data of TD children are placed in the third, or second quadrant, but very close to the zero axis, meaning a contralateral representation for pSMC and contralateral or bilateral representation for AON, analogously to the dominant hand. For UCP children, the majority of data lies within the first and third quadrants, demonstrating a concordance in lateralization of pSMC and AON, with both representations in the unaffected (first quadrant, numbers 2, 8, and 12) or in the affected hemisphere (third quadrant, numbers 3, 5, 6, 7, 9, and 10). Subject number 11 is in line with the TD group, with contralateral representation for pSMC and bilateral representation for AON, while subject number 1 is the only exception with a shifted reorganization in the contralesional hemisphere for AON (as at TMS), but with a higher activation of ipsilesional pSMC for the sensory-motor task.

## 4. Discussion

This study explores for the first time AON of goal-directed actions in UCP children and its relation to type and timing of lesion, sensory-motor reorganization, and clinical assessment.

The first very relevant finding is that AON in UCP children engages brain activations similar to healthy age-matched children despite the presence of brain damage. However, neural networks activated by UCP children present a higher lateralization of maps with a higher activation of the unaffected hemisphere with respect to bilateral representation in the TD group. In our previous study [5], by comparing AON of TD children with that of healthy adults, we demonstrated that lateralization of AON is age-dependent and that adults have a more lateralized activated network in the dominant hemisphere while healthy children have more bilateral and widespread AON. This early lateralization, as a consequence of reorganization due to brain damage, could be reflective of unknown mechanisms that in turn determine an exclusion of natural development through a bilateral activation phase, affecting functioning in UCP children. Our results seem to confirm this hypothesis, because higher AON lateralization was correlated with lower performances on both scales (MUUL and AHA), while UCP children with bilateral AON activation similar to that of the TD group have better performances, reaching higher MUUL and AHA values (Figure 5). This finding is in line with examples of maladaptive plasticity in the context of reorganization of the sensory-motor system where contralateral (ipsilesional) reorganization is more effective in restoring good motor function as opposed to ipsilateral (contralesional) reorganization which is associated with lower grasping and manipulation skills [6, 7].

Another possible explanation could be related to the crucial role of the mirror neuron system in early motor learning by facilitating associations between action perception and corresponding motor programs [27–29]. An altered functioning of imitation capabilities, associated with limited motor system functioning, in early infancy, could contribute to determining maladaptive plasticity.

Regarding properties of observed actions, we found a significant “hand identity” effect in AIP of the hemisphere contralateral to the nondominant hand, similar to TD children [5]. However, contrarily to age-matched controls, this area did not present a significant effect for the “complexity” factor. Lack of significance for “complexity” could be related to the fact that all observed actions may be viewed as complex for UCP children. Another possible explanation could be that AON of UCP children processes mainly for goals rather than kinematics. These explorative hypotheses need further investigation.

For the first time, this study assessed in the same group of subjects both AON and SMN by using fMRI and correlated results with TMS data. Regarding fMRI results, we found a congruence between activation of contralateral pSMC during execution of the sensory-motor task and bilateral or contralateral activation of AON.

Moreover, a good correspondence was also found between AON and TMS data. In particular, observation of the nondominant hand elicited a greater activation of the affected hemisphere in children with ipsilesional reorganization at TMS, while children with contralesional reorganization at TMS had generally either bilateral activation or higher activation in the unaffected hemisphere (Figure 6(b)). However, relevant discrepancies between fMRI results of SMN and reorganization, measured by TMS, were found in two children (numbers 1 and 3, with type I lesion, Figure 6(c)) who presented a higher activation of pSMC of the affected hemisphere despite a TMS reorganization shifted to the contralesional hemisphere. The lateralization index for SMN should be driven by prevalence of sensory contribution to the activity in pSMC of the ipsilesional hemisphere, as confirmed by an explorative post hoc analysis of fMRI data for the sensory-motor task of the nondominant hand, using different regressors for sensory and motor blocks. Considering also clinical performances of the two patients, this finding could be related to a different reorganization of the motor and sensory system and to possible sensory-motor dissociation [6].

Concerning relationships between lateralization index, type of lesion, and type of reorganization evaluated with TMS, all type I UCPs have an ipsilateral (contralesional) reorganization, while type II and III UCPs are variable with lower abilities in children with ipsilateral (contralesional) reorganization and higher abilities in those with contralateral (ipsilesional) reorganization (Table 1). This finding is in line with current literature. In addition, this study shows that type I presents lower clinical scores than the other types do and higher values of lateralization indices, and type II has higher clinical scores and LI values similar to the TD group (bilateral representation), while type III has very high variability. In particular, there are two subjects with similar intermediate MUUL values but opposite LIs, one being more lateralized to the contralateral hemisphere and the other to the ipsilateral one. These diverse findings are related to the different clinical features of these two cases. In the child (number 10) with preferred AON lateralization in the affected hemisphere (LI < 0), also SMN activation and reorganization at TMS were present in the affected hemisphere and her functional level at HFCS was very good. On the contrary, the other child (number 12), with a lower level at HFCS, had greater activations in the contralateral unaffected hemisphere for AON and pSMC, in accordance also to reorganization at TMS.

Another interesting result is that observation of the dominant hand induced a higher, but not significant, activation of the contralateral hemisphere in UCP than the TD group (Figure 4(a)), suggesting a more lateralized circuit in UCP children. This is indirectly confirmed by observation of the nondominant (affected) hand which showed similar LIs between the two groups: although with bilateral activations, the UCP group showed higher variability among subjects, including single cases with high absolute LI values, indicating a specific lateralization in one of the two hemispheres (Figure 4(b)). This finding is in contrast with results of another study in which, for either side, observation of hand movements recruited the primary motor cortex contralateral to the viewed hand, while observation of the paretic side activated more strongly ipsilesional pSMC than viewing movement performed by the nonparetic side [17]. Moreover, in the same study, an engagement of AON was revealed regardless of degree of motor impairment assessed by a hand motor function Likert scale (1–4). These different findings could be related to a difference in the employed paradigm and in particular to non-goal-directed simple hand movements in the allocentric (third person) perspective of that study. Conversely, in the present study, simple and complex goal-directed movements were presented in the egocentric (first person) perspective. Concerning the issue of perspective from which action is observed, in monkeys it has been demonstrated that when comparing neural activation due to different points of view, such as first-person or third-person, first-person might be preferred [30]. An fMRI study in healthy adults showed that while the first-person perspective elicits activations in the hemisphere contralateral to the performing hand as if modelled action was mimicked with the same anatomical hand, in the third-person perspective, parietal activation ipsilateral to the modelled hand was found, indicating a specular strategy, rather than anatomical reproduction [31].

Moreover, the lack of correlation with degree of motor impairment in the study of [17] could be related to the narrower range of the Likert scale with respect to MUUL and AHA. The relationship between MUUL scale and type of sensory-motor reorganization has been previously reported [6], and both MUUL and AHA are highly correlated to lesion extension [32]. We have shown that not only the type of reorganization assessed with TMS but also the laterality index of AON (ALL TASKS > BASELINE) is related to MUUL and AHA scores if we consider type I and type II brain lesions (Figure 5). A similar result was found considering observation of actions performed by the nondominant hand ((C-ND + S-ND) > BASELINE); however, the lack of significance (*p* = 0.07) could be due to high variability of type III lesion (e.g., similar MUUL values with different types of reorganization and opposite laterality indices values).

As far as the relationship between SMN of each hand and laterality index is concerned, we have shown that in the TD group, beyond dominance, there is activation of pSMC of the contralateral hemisphere, reaching values of complete lateralization. In the UCP group, the dominant/unaffected hand induces similar activations while the nondominant/affected hand induces very variable activation with values varying from activation of the contralateral (lesioned) hemisphere to prevalent activation of the ipsilateral, unaffected hemisphere (Figure 4(d)). Another important finding is the lack of relationship between LI values of the nondominant hand and clinical scales, in contrast to the previous interesting relationship of clinical scales with LI of AON. This finding is in accordance with previous studies [33], and it could be related to different reorganization patterns among subjects that determine a huge variability of data with widespread values. All enrolled subjects did not undergo any intensive treatment for the upper limb, and from literature it seems that intensive treatments induce higher LI values with more lateralization in the affected hemisphere [34]. Another possible explanation could be related to the possibility of associated movements (e.g., mirror movements) that can alter SMN data. The solution of excluding UCP children with mirror movements from fMRI studies of SMN is not practicable since it would limit applicability to a small number of subjects. In our sample, the mirror movements, even if assessed with a nonstandardized method [35, 36], were present in 8 out of 12 UCP children. Further studies in UCP children with quantitative and standardized assessment of MM could shed light on the role of MM in SMN reorganization and clinical outcome.

Moreover, the sensorimotor tasks, especially for the affected hand, are often challenging for UCP children, and their execution can generate and be accompanied by an excessive head motion during fMRI sessions. In this study, we paid careful attention to the analysis and compensation of motion and we succeeded in obtaining fMRI data from all subjects for AON and from 11 out of 12 subjects for SMN. Taking into account these issues and considering the good concordance of lateralization indexes for pSMC and AON (Figure 7), the paradigm for exploring AON seems more reliable for studying the motor system in all UCP children, due to its greater feasibility. In fact, for AON tasks, children must only observe actions without doing any physical movement. Plasticity of the AON system with respect to the sensorimotor system still requires greater investigation.

## 5. Conclusion

This fMRI study explores, for the first time, AON of goal-directed actions and SMN in UCP children and their relation to type and timing of lesion, sensory-motor reorganization (TMS), and clinical assessment.

A good congruence was found between bilateral or contralateral activation of AON and SMN activation, TMS data, and clinical scores, suggesting that our paradigm might be useful in exploring AON and adaptive mechanisms or maladaptive plasticity. All these results, based on a small and variable group of UCP children, are necessarily exploratory and need to be extended to and confirmed by other studies. However, collectively, they indicate that, despite congenital and large brain lesions, AON is very active in these children, although with some characteristic differences when compared to TD children. These findings support clinical trials that have been carried out and are in line with numerous ones in progress using action observation therapy (AOT) as a tool to improve manual function, also in chronic phases of these children. Our explorative attempts to correlate manual proficiencies as shown by clinical scales and fMRI, TMS, and other findings may indicate ways to explain and predict efficacy of rehabilitation in UCP children. The fMRI paradigm could also be particularly suitable to investigate effects of plasticity induced by this specific rehabilitation program [37–39].

## Figures and Tables

**Table 1:** Demographic and clinical data of children with UCP enrolled in fMRI study.

ID	Sex	Age (y)	Type of lesion	Side of hemiplegia	Type of reorganization	HFCS	MUUL	AHA	MM
1	M	7.5	I	RH	CL	5	72.13	65.15	Y
2	M	11.0	I	LH	CL	4	60	47	Y
3	M	11.5	I	LH	CL	5	74.59	60.61	Y
4	F	16.3	I	LH	CL	4	55	42	Y
5	M	8.1	II	RH	IL	7	90.98	86.36	N
6	M	10.6	II	RH	IL	8	93.58	77.27	N
7	F	12.0	II	LH	CL	5	79	53	Y
8	M	13.6	II	LH	CL	5	79.51	56.06	Y
9	F	6.2	II	RH	IL	6	81.15	63.64	Y
10	F	7.2	III	RH	IL	8	81.15	69.7	N
11	M	9.3	III	LH	IL	7	91	80.3	N
12	F	10.6	III	RH	CL	5	80.32	75.76	Y

F: female; M: male; y: years; I: early malformative; II: prenatal; III: connatal; RH: right hemiplegia; LH: left hemiplegia; IL: ipsilesional; CL: contralesional; HFCS: house functional classification system; MUUL: Melbourne Assessment of Unilateral Upper Limb Function; AHA: Assisting Hand Assessment; MM: mirror movements; Y: present; N: absent.

**Table 2:** Areas elicited by observation of all object-related hand actions versus BASELINE condition in UCP children and age-matched TD children [5].

Area name	BA		UCP children					TD children				
			Talairach's coordinates			Cluster size	*t*-value	Talairach's coordinates			Cluster size	*t*-value
			*x*	*y*	*z*			*x*	*y*	*z*		
Inferior temporal gyrus	37	DS	−44	−65	0	8307	18.4	−44	−64	0	15,536	20.6
		Non-DS	47	−61	−2	8369	15.6	44	−61	1	18,659	18.7

Superior temporal gyrus	22	DS	−48	−35	12	1357	10.4	−51	−40	11	1531	8.2
		Non-DS	53	−33	9	1534	8.6	50	−40	11	2584	9.2

Inferior parietal lobule	40	DS	−44	−40	39	4278	7.6	−56	−31	30	3751	9.9
		Non-DS	48	−36	36	2281	7.7	57	−31	27	1669	6.9

Anterior IPS	40-7	DS	−36	−45	45	4231	12.7	−32	−44	50	4806	15.4
		Non-DS	37	−50	48	3023	7.3	33	−45	49	2265	10.6

Superior parietal lobule	7	DS	−16	−66	45	5112	11.9	−25	−67	46	11,593	15.7
		Non-DS	22	−62	45	3212	8.0	23	−64	47	8594	11.7

Precentral gyrus	6-4	DS	−35	−11	51	1688	6.2	−33	−16	52	3292	7.9
		Non-DS	35	−9	53	1458	7.2	32	−14	52	1865	5.3
	6-9	DS	−40	4	38	1260	8.3	−47	−2	32	2019	9.5
		Non-DS	43	6	39	1808	7.1	43	2	34	3242	9.7

Middle-superior frontal gyrus	9-10-46	DS	−50	14	24	925	5.0	−39	38	22	1180	5.0
		Non-DS	41	23	17	899	7.2	43	17	21	1154	5.1

Inferior frontal gyrus	45–47	DS						−33	23	2	441	5.2
		Non-DS	45	32	5	982	5.1	41	23	3	538	6.2

Middle occipital gyrus	18	DS	−23	−85	4	1465	10.3	−22	−83	3	3897	11.6
		Non-DS	22	−84	7	1417	7.4	24	−83	2	1369	14.9

BA = Brodmann area; UCP = unilateral cerebral palsy; TD = typically developing; DS = dominant side (controlateral to dominant hand); non-DS = nondominant side (controlateral to nondominant hand); IPS=inferior parietal sulcus. For convention, the dominant hand corresponds to the unaffected hand in UCP and to the right hand in TD children, while the nondominant hand corresponds to the plegic hand in UCP and to the left hand in TD children.

**Table 3:** Areas elicited by sensory-motor task for the dominant hand (DH) and nondominant hand (non-DH) in UCP children and in age-matched TD children.

		BA		UCP children					TD children				
				Talairach's coordinates			Cluster size	*t*-value	Talairach's coordinates			Cluster size	*t*-value
				*x*	*y*	*z*			*x*	*y*	*z*		
Motor-sensory task of DH	pSMC	2-3-4	CLH	−36	−27	53	8926	16.4	−40	−33	53	8811	17.9
			ILH	37	−29	48	1794	6.1	42	−30	54	1046	5.0
	IPL	40	CLH	−50	−29	26	1404	6.3	−50	−26	23	1212	5.5
			ILH	51	−24	25	1946	5.0	54	−20	22	931	6.0
	PrC gyrus	6	CLH	−56	−3	29	1430	5.1	−57	−4	36	712	5.0
			ILH										
	Insula	13	CLH	−44	−11	16	663	6.2					
			ILH										
	SMA	6–31	IH	−5	−17	48	1565	7.1	−4	−25	47	1024	6.9
	Cerebellum		CLH										
			ILH	14	−52	−23	1243	5.0	20	−52	−28	562	5.0

Motor-sensory task of non-DH	pSMC	2-3-4	CLH	35	−29	49	5928	10.1	38	−31	52	8645	18.3
			ILH	−41	−22	46	1032	7.5	−40	−25	55	621	5.8
	IPL	40	CLH	50	−20	27	1719	6.6	50	−25	22	1168	7.2
			ILH	−50	−29	25	502	5.1	−54	−30	28	1445	6.5
	PrC gyrus	6	CLH	48	3	31	878	5.3	42	−9	43	839	6.6
			ILH	−54	−5	29	554	5.7					
	Insula	13	CLH	33	−24	17	613	5.6					
			ILH	−46	−13	11	677	5.6					
	SMA	6–31	IH	0	−12	47	988	5.0	1	−18	52	788	6.1
	Cerebellum		CLH										
			ILH	−14	−52	−21	1495	7.3	−13	−61	−22	2288	7.0

BA = Brodmann area; UCP = unilateral cerebral palsy; TD = typically developing; pSMC = primary sensory-motor cortex; IPL = inferior parietal lobule; PrC gyrus = precentral gyrus; SMA = supplementary motor area; DH = dominant hand; non-DH = nondominant hand; CLH = contralateral hemisphere; ILH = ipsilateral hemisphere, IH = interhemispheric. For convention, the dominant hand corresponds to the unaffected hand in UCP and to the right hand in TD children, while the nondominant hand corresponds to the plegic hand in UCP and to the left hand in TD children.

## Data Availability

All the data used to support the findings of this study are included within the article.

## Supplementary Materials

Supplementary Materials Figure S1: box plots of LI values obtained by different contrasts in UCP children, grouped according to the absence or presence of mirror movements.
