# Supplementary material for: Reorganization of the Action Observation Network and Sensory-Motor System in Children with Unilateral Cerebral Palsy: An fMRI Study
Source: Neural Plast. 2018 Jul 25;2018:6950547. doi: 10.1155/2018/6950547 (PMC6083552; doi:10.1155/2018/6950547)
Supplement: Supplementary Materials — Figure S1: box plots of LI values obtained by different contrasts in UCP children, grouped according to the absence or presence of mirror movements. [file 6950547.f1.pdf]

Supplementary Materials

Figure S1

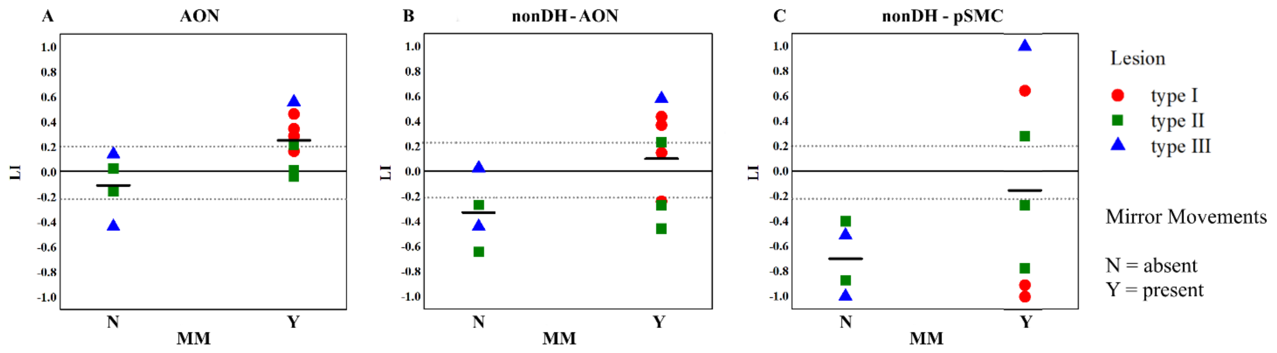

Figure S1: Box-plots of LI values obtained by different contrasts (A. AON task: all TASK>BASELINE; B. AON task: (C-ND + S-ND) > BASELINE; C. Sensory-Motor task: (Sens-ND + Mot-ND) > REST) in UCP children, grouped according to the absence or presence of Mirror Movements (MM, N=absence, Y= presence). As in figures 5 and 6, children were represented with different colours and symbols with respect to the classification of their lesions (Type I = red circles, Type II = green squares, Type III= blue triangles). Grey dotted lines represent the threshold value of |0.20| for hemispheric lateralization.

A. For TASKS>BASELINE contrast of AON, children without MM had bilateral AON activation (LI_N=-0.11±0.25), except for one case (#10). On the contrary, children with MM presented either bilateral activation or higher activation in the hemisphere contralateral to dominant hand (LI_Y=0.25±0.21). The difference was statistically significant (p=0.02).

B. Both lateralization indices decrease accordingly when the previous analysis was performed considering only observation of non-dominant hand: LI_N=-0.33±0.28 for MM-N and LI_Y=0.10±0.38 for MM-Y (p=0.05, Mann-Whitney U Test).

C. Regarding SMN of non-dominant hand, UCP children presented different trends of LI values of pSMC, with respect to absence/presence of MM even if globally their differences were not statistically significant (p=0.24). Children without MM showed a greater activation of pSMC in affected hemisphere (LI_N=-0.70±0.29). Children with MM not present a common behaviour, but rather a certain degree of variability (LI_Y=-0.15±0.80).
